# 

**Published:** 1912-01-27

**Authors:** 


### Buildings Contemplated and in Progress.

Bolton.—Architects are invited to submit competitive designs for a Nurses' Home at the Infirmary. Mr. John B. Cass, F.R.I.B.A., is the assessor; premiums of £30, £20, and £10 are offered, and designs have to be delivered before February 3.

Hastings.—The East Sussex Hospital competitive designs had to be delivered before December 30, 1911, and the award will shortly be made by the assessor, Mr. E. T. Hall, F.R.I.B.A.

Limerick.—Mr. W. Rosengrove is architect for the proposed additions to the Infirmary, estimated to cost £2,500.

Petersfield.—Mr. E. Arden Minty, F.R.I.B.A., is architect for the Petersfield Cottage Hospital extension, which was formally opened a few days ago. It contains two wards for paying patients, and is to be known as a King Edward Memorial.

Skegness (Lincs).—Mr. F. J. Parkinson, of Blackburn, is architect for a cottage hospital to cost about £3,000.

South Elmsall.—At South Elmsall, near Doncaster, a cottage hospital has been built and formally opened by the Carlton Main Colliery Co., for the use of miners at the Frickley Colliery. The new institution is named the "Warde Aldam Cottage Hospital." The total cost, including furnishing, is about £6,000. Illustrations were published in our columns on October 7, 1911.

Stirling.—£12,000 is the estimated cost of the extension to Stirling Infirmary, now being executed. Of this sum £10,628 is in hand.

Wellington, Shropshire.—Under the terms of the will of the late Mrs. Bowring, of Bradeley Moor, Wellington, a piece of land of about three acres in extent was left upon trust for building thereon a cottage hospital, together with a sum for the building, furnishing, and equipment. The competitive designs for the hospital have now been received by the Trustees, and submitted to Mr. W. L. Bernard, F.R.I.B.A., of Bristol, the assessor appointed, who has awarded first place to those prepared by Mr. Leslie T. Moore, A.R.I.B.A., of Raymond Buildings, Gray's Inn, W.C. Mr. Moore has designed more than one Cottage Hospital, his sketch for the latest of which, that at Axminster, was published in The Hospital of November 11, page 155.

Hospital Designs in Montevideo.—The British Hospital in Montevideo, which is supported by voluntary contributions and is much appreciated by the British residents and the employees of the trading companies, as no conditions are made as to the nationality of its patients, is erecting new buildings from the design of Mr. John Adams, of Montevideo. His Majesty's resident minister, Mr. R. J. Kennedy, C.B., is the chairman of committee. The new buildings are expected to cost £16,000. Three male wards are provided for 22 beds, and one six-bed female ward. The first floor provides several private wards besides six single-bed wards on the ground floor and four double-bed wards. An operating room is provided and comprehensive administration apartments. The laundry and post-mortem chambers are placed in an isolated building connected by a covered way. Reinforced concrete foundations are provided and the structures generally are fireproof. The various buildings are much more closely set together than is usual in England, but the stronger light of the semi-tropics makes this permissible.

### Gifts and Bequests.

The King has sent a present of old linen to the Chelsea Hospital for Women.

Mr. J. Doyle Field, of Maldon, has left £250 to the Chelmsford Infirmary.

Mr. Charles Wardlaw, of Sheffield, has left £500 to the Royal Hospital, Sheffield.

Two recent gifts to the North Devon Infirmary have been £100 from Mr. J. Q. Tamlyn and £33 from Mr. Kent, of Tawstock.

Sir Charles Gold, late M.P. for Saffron Walden, has presented to the Bishop Stortford Hospital a donation of £1,000, as a memorial to the late Lady Gold.

Mr. Joseph Withers, of Stratford, E., the founder of a ward in the West Ham Hospital, has bequeathed £20,000 to that hospital and £5,000 to the Victoria Park Hospital.

The Rev. Cecil Henry Maunsell, of Kettering, has left £1,000 to the Kettering and District General Hospital, £500 to the Northampton General Hospital, and £500 to St. Mary's Hospital, Brighton.

Mr. Nathaniel Cohen has bequeathed £100 each to the Great Ormond Street Children's Hospital, to St. Thomas's Hospital, the London Hospital, the London Fever Hospital, Islington, Charing Cross Hospital, Guy's Hospital, St. Bartholomew's Hospital, St. Mary's Hospital, and the Middlesex Hospital.

The London Homœopathic Hospital, Great Ormond Street, Bloomsbury, W.C., has received from a private donor, through the Hospital Saturday Fund, a gift of thirteen large turkeys. Such gifts of provisions, it is understood, are most welcome, as they make a change in the diets and economise the provisions bill.

From the estate of the late Mrs. C. M. Barnato the following grants have been made: £1,000 to the Middlesex Hospital, to the special ward for women and children; £250 to the City of London Lying-in Hospital; £100 to the Hospital for Incurables, Putney Heath; and £50 to the Belgrave Hospital for Children.

Mr. Isaac Gilman, of Warrington, Lancs, has left £1,000 each to the Warrington Infirmary and to the Manchester Royal Infirmary; and, subject to a life interest, a further sum of £2,000 to the Warrington Infirmary, and also £1,000 to the Altrincham Dispensary and Hospital, while the ultimate residue is left to St. Mary's Hospital, Manchester.

Mr. John Roderick, of Handsworth, has bequeathed £50,000 to the Birmingham General Hospital, £10,000 to the Queen's Hospital, Birmingham, and £1,000 each to the General Dispensary and to the Skin and Urinary Hospital, Birmingham. In addition the residue of the estate, amounting to over £150,000, will be divided between the General Hospital, the Dispensary, and the Queen's Hospital; the first-named to receive one-half, and the others a quarter each.

Mr. Alfred John Heath has left a sum sufficient to endow a bed in each of the following hospitals, to be named "The Alfred and Clara Heath Bed": The London Hospital, Charing Cross Hospital, King's College Hospital, Middlesex Hospital, St. Mary's Hospital, the Fulham Road Cancer Hospital, and the Miller Hospital, Greenwich. To the Children's Hospital, Great Ormond Street, a cot has been endowed, to be called the "Cissy" cot. The remainder of his property Mr. Heath has left to the King Edward Hospital Fund.

Mr. John Bowly has left £100 to the Whitehaven and West Cumberland Infirmary.

Miss H. Helah Sutcliff has left £500 to the Grosvenor Hospital for Women and Children.

The Royal Hospital for Incurables, Putney Heath, has received 50 guineas from the Clothworkers Company.

Mrs. Douglas Henty, of Chichester, has given £1,000 towards the building of a new laundry at the Chichester Infirmary.

Mr. Edwin Smith has left 100 guineas each to the Middlesex, St. George's, University College, and the St. John and Elizabeth Hospitals.

Mr. James Wilcock, of Blackburn, has bequeathed £2,000 each to the Blackburn and East Lancashire Infirmary and to the Blackburn District Nursing Association, and £4,000 to the Imperial Cancer Research Fund.

Sir Ernest Cassel has given £2,000 through King Edward's Hospital Fund to King's College Hospital, and £800 to the Great Northern Central Hospital, with a direct donation of £250 in memory of Mrs. Ashbey, his daughter.

Under the will of the late Sir Cuthbert Quilter the Suffolk County Hospital, Ipswich, will receive £500, and the Bury St. Edmunds Hospital, the St. Leonards Hospital, Sudbury, and the Convalescent Hospital, Felixstowe, will receive £100 each.

A Windfall for Bristol Hospitals.—Mr. James Lawes Perrin has bequeathed £500 each to the Bristol Royal Infirmary, the General Hospital, the Dispensary, the Hospital for Women, and the Eye Hospital, Bristol; £500 to the Royal Ear Hospital, Soho Square, and £500 to the Swansea Hospital.

### Florence Nightingale Memorial.

We have received the following contribution: Dr. Wardrop Griffith, £1 1s.

Received by Mr. G. Q. Roberts: Mrs. Charles Heywood, £15; the Incorporated Belfast Nurses' Home, £10; A. L., £5; B., £5; Society for Providing Nurses for the Poor, Belfast, £2 7s. (annuities); Mrs. Crawley, £2 2s.; Miss Anderson's Fiji Collection (additional), per B. Glanville Corney, Esq., £1 2s. 6d.; H. Y. L. Smith, Esq., 10s.; Anon., 10s.; Edward Bamford, Esq., 5s. 4d.; per Mr. Edwin Hodder, 2s.; the Misses Channell (annuities), 2s.

### Appointments.

Mr. H. F. Rutherford, who has held the appointment of out-patient clerk and assistant in stores at Addenbrookes, has been appointed to succeed Mr. H. F. J. Mann.

Mr. Rawdon A. Veale, B.A., M.D., B.S., M.R.C.P., has been elected an Assistant Physician, and Edward W. Bain, M.B., B.S., F.R.C.S., an Aural Surgeon to the Leeds General Infirmary.

Dr. Arthur Hall has been elected Honorary Physician to the Sheffield Royal Hospital for seven years. Mr. F. G. Mordaunt, L.D.S., and Mr. Frank Harrison, L.D.S., Honorary Dental Surgeons for three years; Mr. W. J. Law, L.D.S., Honorary Assistant Dental Surgeon for three years. Mr. Percy S. Stokes, L.D.S., was promoted from Assistant to Honorary Dental Surgeon for three years in the place of Mr. Charles C. Drabble, L.D.S., resigned.

### The Week's Novelties.

### TWO PERFECT WHISKIES.

(A. Dewar Rattray, 188 Dumbarton Road, Partick, Glasgow.)

In a former issue (The Hospital, March 25, 1911) we referred to the "Hero Brand Whisky" manufactured by this firm, which we characterised as a mild, well-matured whisky. Through the courtesy of Messrs. Rattray we have since been favoured with samples of their twenty-year-old and twenty-year-old (Blend Finest Glenlivet) whiskies, respectively sold at 4s. 6d. and 5s. 6d. per bottle. Both these whiskies may be considered as near perfection as it is possible for a liqueur whisky to be. Both are mild in taste, bland, with that smoothness that is as near oiliness as it is justifiable to expect in a liqueur which has not been "smoothed" by the addition of syrup. We have tested the two qualities against similar priced liqueur whiskies of other firms, and have no hesitation in saying that we prefer the "Hero" brand. The blended Glenlivet especially has a fine aroma, which materially enhances the pleasure which is to be derived from slowly sipping a small glass of the liquor. As a flavouring for invalid jellies and as an addition to an egg posset these two liqueur whiskies are cordially to be recommended to hospital cooks. The physician who requires a good wholesome whisky, both for his own table and to recommend to his patients, cannot do better than place his order with Messrs. Rattray. He may rely upon obtaining pure, unadulterated whisky of sterling quality.

### COAL STRIKES AND HOSPITAL FIRES.

It is probably true that the recent panic of a coal strike has been engineered by interested parties, and that hospitals have placed their contracts before prices rose. Nevertheless the price of gas, as quoted by the Gas Light and Coke Company, has fallen to 2s. 6d. per 1,000 cubic feet, and the company claims to hold good stocks of coal. The obvious convenience of the gas fire has, therefore, at the moment certain adventitious advantages to recommend it.

### AROMATIC SCHNAPPS.

(A. C. Nolets, Schiedam.)

The sample of gin forwarded to us by Messrs. A. C. Nolets, of Schiedam, is a pure "Geneva" of fine flavour and body and possessing a peculiar aroma of its own. As a medicinal gin its carminative and diuretic properties should make it a valuable adjuvant in the treatment of cases where this remedy is indicated. As a table or after-dinner drink it will be popular by reason of its bland taste, which is entirely without the bitter and repellent after flavour so often found in the more common brands of gin. The percentage of essential oils in the sample sent to us was higher than in ordinary aromatic gins, but no impurity could be detected. The schnapps is an excellent specimen of its kind, and as such fully worth a trial.

Bovril's bonus pictures are again offered to those who send the coupons that accompany the bottles up to the value of one guinea to the firm before June 29, 1912. One picture, with a romantic historical setting, is called "The Pilgrims," by A. A. Dixon. Two others are a salon picture, "The Idyll," by Paul Gervais, and the "Joy of Spring," by Arthur Elsey, all of which have been reproduced by C. W. Faulkner and Co.

### The Hospital.

Rates of Subscription (payable in advance).

UNITED KINGDOM.

| | | |
|---|---|---|
| Three Months | (including Postage) ... ... ... ... | 2s. 0d. |
| Six Months | do. ... ... ... ... | 4s. 0d. |
| Twelve Months | do. ... ... ... ... | 6s. 6d. |

FOREIGN AND COLONIAL.

| | | |
|---|---|---|
| Three Months | (including Postage) ... ... ... ... | 2s. 6d. |
| Six Months | do. ... ... ... ... | 5s. 0d. |
| Twelve Months | do. ... ... ... ... | 8s. 8d. |

This form may be sent to any Newsvendor, Railway Bookstall, or to The Manager, "The Hospital," 28/9 Southampton Street, Strand, W.C.

*SUBSCRIPTION FORM.*

*Please send me* "The Hospital" *for* *months, commencing with your issue of*........................., *for which please find enclosed*..........................

*Name*...........................................

*Address*........................................

...........................................

Date..........................................

*To*..................................*Newsagent,*

### SANATORIA FOR THE PEOPLE.

**1/- net.** The State Campaign Against Tuberculosis. **1/- net.**

CONTENTS.

**What is a Sanatorium?**
**The Sanatorium as the Training School of Health.**
**The Relation of Sanatoria to other Methods.**
**Consumption as an Industrial Disease.**
**Our Present Consumption Bill.**
**The After Care of Sanatorium Patients.**
**What the Insurance Bill can Effect.**

In addition to covering the ground set out above, the volume compresses into its small compass a study of Consumption as an Industrial disease, founded on new figures and facts which have hitherto in many instances been unavailable to the public. Crown 8vo, cloth. 1/- net. 1/2 post free.

**COTTAGE HOSPITALS, GENERAL, FEVER AND CONVALESCENT.** Their Progress, Management and Work in Great Britain, Ireland, and the United States of America. By Sir HENRY BURDETT, K.C.B., K.C.V.O. Third Edition, profusely Illustrated, with nearly 50 Plans, Diagrams, &c. Crown 8vo, cloth gilt. **10/6** Post free.

**HOSPITAL EXPENDITURE: THE COMMISSARIAT.** For Hospital Matrons and Managers. Crown 8vo, cloth. **2/6** Post free.

**A LEGAL HANDBOOK FOR THE USE OF HOSPITAL AUTHORITIES.** By LEONARD SYER BRISTOWE, M.A.Oxon., Barrister-at-Law. Crown 8vo, cloth. **2/6** Post free.

**THE MEDICAL ATTENDANCE OF LONDONERS.** An Economical System of Medical Relief freed from existing abuses and adequate to protect all classes and interests. By Sir HENRY BURDETT, K.C.B., K.C.V.O. **1/-** Post free.

**FURNISHING AND APPLIANCES OF A COTTAGE HOSPITAL.** An Actual Inventory. By the Hon. SYDNEY HOLLAND. Second Edition, paper cover. **6d.** net, **7d.** Post free.

**THE COTTAGE HOSPITAL.** Its Origin, History, Value and Advantages to all Residents in Rural Districts. Being a Speech delivered at Welwyn, Herts. By Sir HENRY BURDETT, K.C.B., K.C.V.O. **6d.** each, **30/-** Per 100, Post free.

**SURGICAL WARD-WORK AND NURSING.** By ALEXANDER MILES, M.D.Edin., C.M., F.R.C.S.E. Second Edition, enlarged and entirely Revised. Demy 8vo, copiously illustrated, cloth gilt. **5/-** net, **5/5** Post free.

**A MANUAL OF ABDOMINAL SURGERY FOR NURSES.** By HAROLD BURROWS, M.B., F.R.C.S. Crown 8vo, cloth, illustrated. **2/6** net, **2/9** Post free.

**SURGICAL INSTRUMENTS AND APPLIANCES USED IN OPERATIONS.** A Classified and Illustrated List, with Explanatory Notes. By HAROLD BURROWS, F.R.C.S. Third Edition, Revised and Enlarged, with special new chapter on Sterilisation of Instruments. Crown 8vo, limp cloth. Entirely new Illustrations, specially drawn. **1/6** net, **1/8** Post free.

**THE EXAMINATION OF THE URINE.** By J. K. WATSON, M.D., M.B., C.M., Author of "A Handbook for Nurses." Demy 16mo, cloth boards. **1/-** net, **1/1** Post free.

